# Behavioural Response to the Environmental Changes of Various Types in Lister-Hooded Male Rats

**DOI:** 10.1038/s41598-019-42924-1

**Published:** 2019-05-08

**Authors:** Wojciech Pisula, Klaudia Modlinska, Anna Chrzanowska

**Affiliations:** Institute of Psychology, Polish Academy of Sciences, Warsaw, Poland

**Keywords:** Psychology, Behavioural ecology

## Abstract

The animal preference for complexity is most clearly demonstrated when the environmental change takes the form of an increase in complexity. Therefore, one of the potential difficulties in interpretation is that the preference for perceptual novelty may be confounded with the change in environmental complexity. In this study, the environmental complexity was controlled by manipulating with tunnels inside the experimental chamber. Adding new tunnels triggered a very profound change in behaviour, which was demonstrated by the animals’ prolonged stay in the proximity of the novel objects, sniffing, touching, and climbing on top of the tunnels. The removal of the tunnels from the test arena turned out to have the least influence on behaviour compared to the other manipulations used in this study. The reduction of complexity of the tunnels had a moderate effect on rat behavior. Tunnels are important elements in the rats’ environment, since they provide various possibilities for hiding, resting or moving inside the tunnel. They may be treated as a good example of affordances in rat-environment interactions. The results of this study may therefore serve as a basis for constructing a modified theory of animal curiosity which could incorporate the concept of ecological psychology.

## Introduction

Environmental complexity is one of the key characteristics of the surrounding environment. Complexity may be understood as affordances, that is, the available forms of behaviour that a particular environment offers the individual^1^ and the extent to which these forms contribute to regulating the interaction between the environment and specific behaviours. At the operational level, affordances take the form of objects provided by the environment which create opportunities to activate certain behavioural patterns in response to those objects.

At the biological level, changes in behaviour occurring due to increased environmental complexity have been linked to various changes in the brain^2^. The crucial role of the environmental complexity for the central nervous system, as well as behavioural development, has also been widely documented^3–9^. In addition, environmental complexity increases novelty-seeking behaviour^10^ and object exploration^11^, while reducing anxiety-like behaviour and increasing activity^12^.

It is well known that animals prefer more complex environments rather than simplified or impoverished ones^13–15^. This preference for complexity is most clearly demonstrated when the environmental change takes the form of an increase of complexity. This phenomenon has been consistently observed in our laboratory^16–20^. What is more, laboratory rats show a positive response to low-stress or non-stressful novel events^16–20^. This finding supports the claim that the rewarding aspects of novelty play an important role in regulating animal behaviour.

In research literature, novelty is usually achieved by introducing a novel object or exposing the animal to novel stimuli, which increases environmental complexity (e.g.^21,22^). This subsequently affects the number and quality of possibilities and/or affordances available to the animal^23^. Moreover, in most studies, environmental change involves introducing a novel object into a familiarised environment. For this reason, one of the potential difficulties in interpretation is that the preference for perceptual novelty may be confounded with the change in environmental complexity. Thus, in order to fully understand the nature of the response to novelty which results from a change in the stimulus field, it is crucial to determine the extent to which the positive response to novelty is compounded with the shift in the animal’s preference towards more complex environments.

It may be suggested that if the magnitude of change, but not its direction, triggers exploratory behaviour, both a decrease and an increase of complexity will have similar effects on the subjects. However, if stimulus complexity plays a role in regulating the adaptation to novelty^24,25^, we may expect differential responses to changes caused by an increase and decrease of complexity, respectively.

The purpose of this study was to further explore the responses to novelty which are triggered by changes in the stimulus field. It was designed to assess exploratory behaviour in response to environments of increased and decreased complexity. Both types of environmental changes introduce novelty into the environment. The experimental chamber used in this study had already been proved to create appropriate conditions for measuring exploratory activity in rats^16–20^. In this study, environmental complexity was achieved by placing tunnels inside the experimental chamber. The tunnels were manipulated so as to decrease and increase environmental complexity. At the operational level this meant that the tunnels were alternately removed and replaced, and that the environmental complexity was decreased without any changes to the dimensions of the objects.

## Methods

### Animals

The sample consisted of 44 experimentally naive male Lister Hooded, outbred rats. The animals were sourced from Charles River, Germany, via AnimaLab Sp. z o.o., Poland. The rats were housed in the vivarium of the Institute of Psychology, Polish Academy of Sciences, Warsaw, Poland. The rats were approx. 90 days old and weighed approx. 280 g at the start of the experiment.

The rats were housed in groups of 3–4 in Tecniplast© Eurostandard Type IV cages (610 mm × 435 mm × 215 mm) with dust-free softwood granules Tierwohl Super© as bedding and with ad libitum access to water and standard laboratory fodder (Labofeed H, WP Morawski, Kcynia, Poland). The day/night cycle was set at 12/12 h, with the lights-on at 8 AM, and the temperature was maintained at a constant 21–23 °C. The cages and pens were cleaned once a week, on the same day and at a fixed time, in the late afternoon (5 pm). However, in order to ensure that the experimental procedure was not disrupted, the cages in which the test animals were kept were cleaned just before the onset of the experiment and again after the experiment was finished. All the rats kept in our laboratory were housed, bred and taken care of in accordance with the Regulation of the Polish Minister for Agriculture and Rural Development of 14 December 2016 on laboratory animal care; the experimental procedures had been approved by the 1st Local Ethics Committee on Animal Experimentation in Warsaw, Poland. The sample size was estimated using a commonly used formula for calculating sample size for repeated measures^26^:

$${\rm{N}}={\rm{2}}+{\rm{C}}({\rm{s}}/{\rm{d}}){\rm{2}}$$

where: s – standard deviation of the population means. d – size of difference in means (the effect). C – constant dependent on the value of α (significant level) and 1-ß (power). For the purpose of our study, we employed the following parameters: α = 0.05; β = 0.20 (C = 10.51). Group size calculations were carried out on the basis of a previous study^27^ in which the average time spent on exploring changed objects was M = 96.51, with standard deviation s = 37.35, and on the assumption that the detectable difference between the variables should be d = 40. Therefore, the total sample size was estimated at 12.

### Procedure

The aim of the exploration test was to compare the process of investigation of a new environment, the rate of habituation to it, and the response to the introduction of innocuous novelty of low intensity into the well-known context. The apparatus and measurement methods were similar to those used in our previous studies^16–20^.

**Figure 1:**
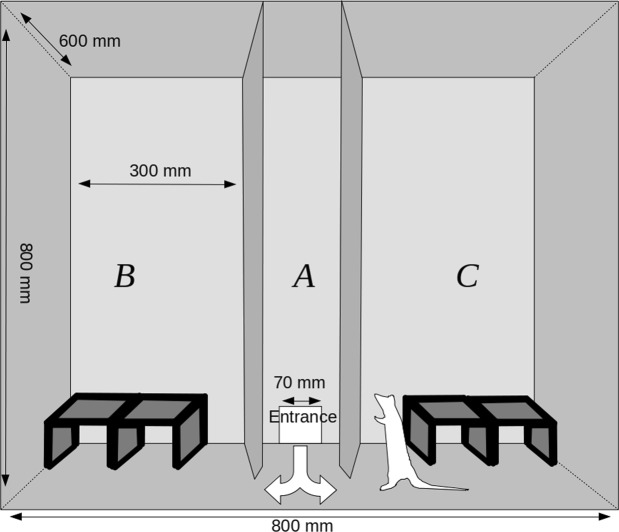
Experimental chamber used for investigating exploratory behaviours.

The experimental chamber (Fig. 1) was a box measuring 800 mm × 600 mm × 800 mm. The chamber was divided into three zones (A, B, and C), which were separated by two walls running perpendicularly to its longer side. The front of the chamber was a transparent wall which could be lifted to gain full access to the experimental arena. The wooden division walls between the zones had triangular entrances (120 mm × 140 mm) at the bottom which enabled the animals to move freely between the chamber parts. The entire chamber was covered with a layer of washable varnish. Wooden tunnels covered with washable paint were placed in zones B and C. The configuration of the tunnels was different in each experimental setting. In contrast to the most frequently used two-dimensional experimental settings, these tunnels provided a complex three-dimensional environment. The central zone (A) was left empty – there was a hole in the back wall of the chamber which served as an entrance for animals moving from the transporter into the chamber.

**Figure 2:**
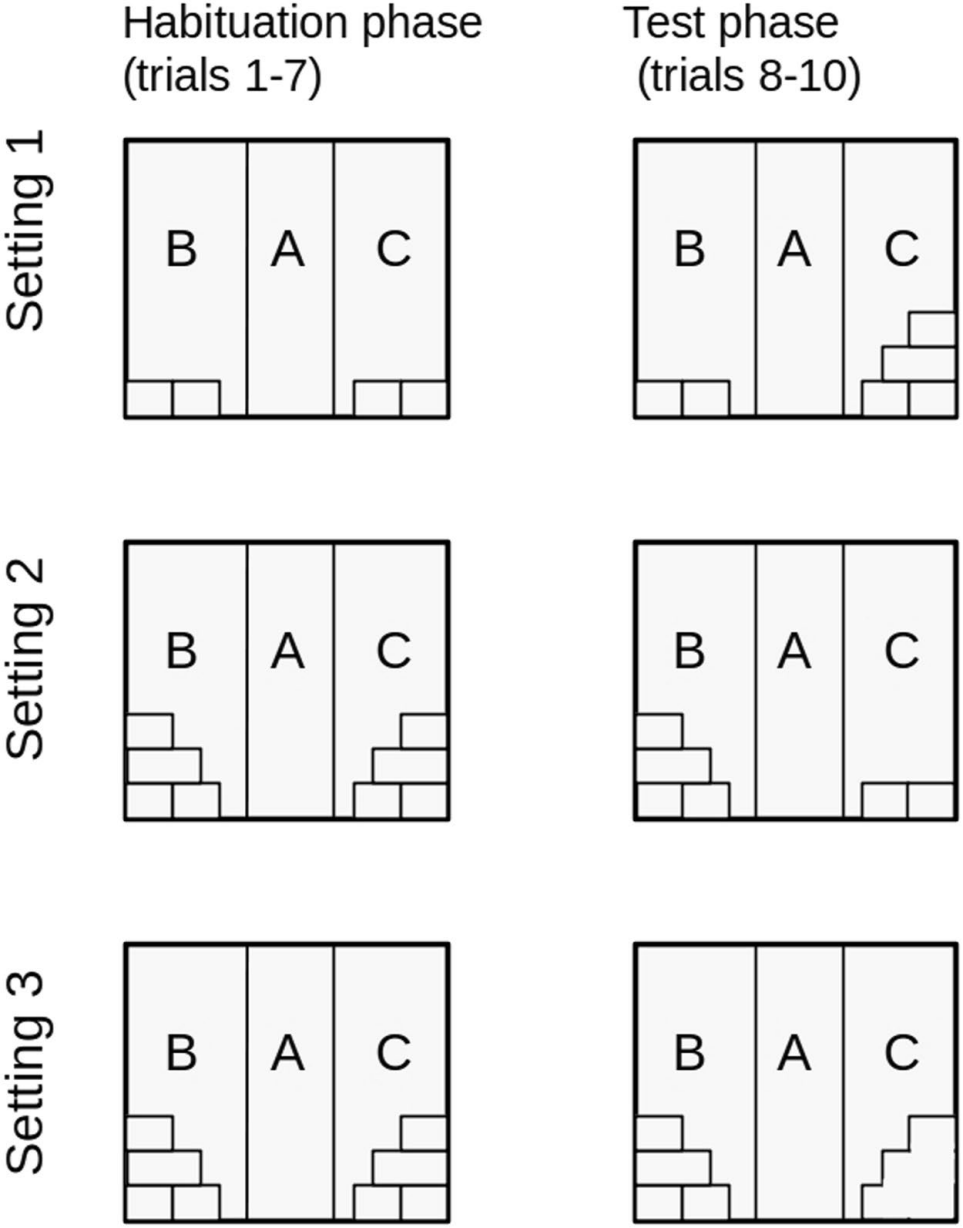
Arrangement of objects in the experimental chamber in each experimental setting.

At the start of each trial, a small cylindrical cage - transporter (the ‘starting box’ – 60 mm in diameter with doors 120 mm high and 100 mm wide) with the test animal inside was placed by the entrance to zone A. Then the rat was left undisturbed in the starting box for 15 seconds, after which the entrance door was opened. The animal was free to stay in the starting box or to leave it to explore the chamber. The first seven trials were habituation trials during which the apparatus was arranged in the same way (Fig. 2). The introduction of novelty took place between trials 7 and 8. Three subsequent trials were conducted with the chamber in this new arrangement (Fig. 2). Each trial lasted 7 minutes and was conducted for each animal once a day. The test sessions began every day at 10 am, i.e. two hours after the onset of daytime. The experimental groups were divided into smaller sub-groups of 3–4 individuals, and the rats from different experimental condition were tested alternately. By proceeding in this way, we attempted to avoid the effect of the specific time of day on the behaviour of animals from particular experimental groups.

After each session, the experimental arena and the starting box (transporter) were thoroughly cleaned with Virkon S (Bayer) in order to remove odour cues left by the previous animal.

A video camera was placed approximately 1.2 m away from the transparent front wall of the experimental chamber. The camera was set in the night-shot mode to enable filming in the dark. Behaviours observed were coded on the basis of the recorded material using BORIS event logging software^28^. This program makes it possible to define particular behaviours and to score the time and frequency of selected behaviours. In this study, we scored selected behaviours occurring during the entire experimental session. As a result, the exact time of individual bouts of behaviours, their frequency and, consequently, the total time spent engaging in a given behaviour were assigned specific scores. The behaviours analysed comprised the following: latency to leave the starting box; amount of time spent in the starting box (excluding the latency to leave the starting box); total time spent in the unchanged zone of the chamber; total time spent in the changed zone of the chamber; time spent on contact with the tunnels in the unchanged zone of the chamber; frequency of contact with the tunnels in the unchanged zone of the chamber; total time spent on contact with the tunnels in the changed zone of the chamber; frequency of contact with the tunnels in the changed zone of the chamber; and frequency of moving between the zones of the chamber.

Three experiments were conducted which differed in the configuration of the tunnels that were placed in the experimental chamber, as well as in the type of novelty provided in the 7th trial. In each experimental setting, the tunnels were placed in zones B and C.

Setting 1 - Addition (ADD) of a novel object to the other objects in the experimental box (the ADD group). During the habituation sessions, two tunnels (200 mm × 120 mm × 80 mm) had been placed in each of the zones B and C and arranged in the same way (Fig. 1). On the first trial day (trail 8), two additional tunnels were put in zone C (Fig. 2). The arrangement of the tunnels in zone B remained unchanged. The ADD group consisted of 13 rats.

Setting 2 - Removal (RMVL) of a familiarized object from the experimental box (the RMVL group).

During the habituation sessions, four tunnels had been placed in each of the zones B and C which had been arranged in three layers (Fig. 2). On the first trial day (trail 8), the two top tunnels were removed from zone C (Fig. 2). No changes were made to the new arrangement in zone C until the end of the experiment. The arrangement of the tunnels in zone B remained unchanged. The RMVL group consisted of 17 rats.

Setting 3 - Reduced (RDCP) complexity of objects (the RDCP group). During the habituation sessions, four tunnels had been placed in each of the zones B and C which had been arranged in three layers (Fig. 2). On the first trial day (trail 8), the tunnels in zone C were replaced with an object of the same size as the tunnels which had been removed but without the partitions (Fig. 2). No changes were made to the new arrangement in zone C until the end of the experiment. The arrangement of the tunnels in zone B remained unchanged. The RDCP group consisted of 14 rats.

To avoid the confounding effect of lateralisation or visual/auditory cues, the novelty was introduced in the left zone (as described above) for half of the rats tested, and in the right zone for the remaining half (a mirror image of Fig. 2).

## Results

To enhance the legibility of the results, graphs and tables, the habituation phase has been indicated as H (mean score from habituation trials 5 to 7, which served as a reference value for further analyses), while the test trials have been indicated as T1, T2, and T3, respectively. Novelty (i.e. addition or removal of tunnels in zone C) was introduced in the first test trial (T1).

We have decided not to present the results of the initial four habituation trials, since they serve only as the habituation phase and not as an element of a comparative analysis of the animals’ response to novelty.

The data was analysed using a General Linear Model procedure (GLM), with repeated measurements (H, T1, T2, T3) as within-subject factors, followed by a LSD PostHoc test, which involved a comparison of the habituation phase (H) with the three test trials (T1, T2 and T3). Differences were considered significant for p ≤ 0.05. Data analysis was carried out using the IBM SPSS software (version 24).

### Latency to leave the transporter

The analysis using GLM for repeated measures showed no significant differences for experimental trial in ADD [F(3, 36) = 1.487, p = 0.235], RMVL [F(3, 48) = 0.907, p = 0.445] and RDCP [F(3, 39) = 0.948, p = 0.427] groups.

### Time spent in the transporter

The amount of time spent in the transporter, excluding the latency to leave the transporter was also measured for each group. In the **ADD** group, the analysis showed a significant main effect of trial: F(3, 36) = 6.640, p = 0.010, Eta^2^ = 0.666 (Wilks’ Lambda). A post-hoc analysis showed a significant decrease in the time spent in the transporter in all test trials compared to the habituation phase (T1: p = 0.003, M_H_ = 63.62, SD_H_ = 19.93, M_T1_ = 36.62, SD_T1_ = 20.54; T2: p = 0.050, M_T2_ = 41.31, SD_T2_ = 26.18; T3: p = 0.004, M_T3_ = 34.54, SD_T3_ = 21.93).

In the **RMVL** group, Mauchly’s test indicated that the assumption of sphericity had been violated (χ^2^(2) = 24.4, p < 0.001), so the degrees of freedom were corrected using Greenhouse-Geisser estimates of sphericity (ε = 0.5). The analysis showed a significant main effect of trial: F(1.494, 23.9) = 4.497, p = 0.031, Eta^2^ = 0.219. A post-hoc analysis showed a significant increase in the time spent in the transporter in third test trial (T3: p = 0.027, M_H_ = 54.63, SD_H_ = 14.84, M_T3_ = 83.59, SD_T3_ = 52.93).

In the **RDCP** group, the analysis showed no significant main effect of trial: F(3, 39) = 2.270, p = 0.137 (Wilks’ Lambda).

### Time spent in the unchanged zone of the chamber

In the **ADD** group, Mauchly’s test indicated that the assumption of sphericity had been violated (χ^2^(2) = 12.3, p = 0.032), so the degrees of freedom were corrected using Greenhouse-Geisser estimates of sphericity (ε = 0.7). The analysis showed a significant main effect of trial: F(2.101, 25.207) = 14.092, p < 0.001, Eta^2^ = 0.540. A post-hoc analysis showed a significant decrease in the time spent in the unchanged zone in all test trials compared to the habituation phase (T1: p < 0.001, M_H_ = 131.54, SD_H_ = 15.83, M_T1_ = 68.00, SD_T1_ = 20.78; T2: p = 0.015, M_T2_ = 99.85, SD_T2_ = 42.38; T3: p < 0.001, M_T3_ = 82.23, SD_T3_ = 37.19).

In the **RMVL** group, the analysis showed no significant main effect of trial: F(3, 48) = 1.104, p = 0.381 (Wilks’ Lambda).

In the **RDCP** group, the analysis showed a significant main effect of trial: F(3, 39) = 6.640, p = 0.024, Eta^2^ = 0.561 (Wilks’ Lambda). A post-hoc analysis showed a significant increase in the time spent in the unchanged zone in the third test (T3: p = 0.027, M_H_ = 122.38, SD_H_ = 39.99, M_T3_ = 163.29, SD_T3_ = 49.29).

### Time spent in the changed zone of the chamber

In the **ADD** group, the analysis showed a significant main effect of trial: F(3, 36) = 26.679, p < 0.001, Eta^2^ = 0.889 (Wilks’ Lambda). A post-hoc analysis showed a significant increase in the time spent in the changed zone in all test trials compared to the habituation phase (T1: p < 0.001, M_H_ = 117.62, SD_H_ = 18.31, M_T1_ = 220.62, SD_T1_ = 38.85; T2: p < 0.001, M_T2_ = 210.85, SD_T2_ = 72.11; T3: p < 0.001, M_T3_ = 231.85, SD_T3_ = 48.37).

In the **RMVL** group, the analysis showed no significant main effect of trial: F(3, 48) = 0.607, p = 0.621 (Wilks’ Lambda).

In the **RDCP** group, the analysis showed no significant main effect of trial: F(3, 39) = 3.557, p = 0.051 (Wilks’ Lambda).

### Frequency of moving between the zones (left/right/transporter) of the chamber

In the **ADD** group, the analysis showed a significant main effect of trial: F(3, 36) = 5.753, p = 0.015, Eta^2^ = 0.633 (Wilks’ Lambda). A post-hoc analysis showed a significant decrease in the frequency of moving between the zones in all test trials compared to the habituation phase (T1: p = 0.044, M_H_ = 14.18, SD_H_ = 2.15, M_T1_ = 12.62, SD_T1_ = 2.75; T2: p = 0.001, M_T2_ = 11.77, SD_T2_ = 1.96; T3: p = 0.022, M_T3_ = 12.38, SD_T3_ = 2.43).

In the **RMVL** group, the analysis showed no significant main effect of trial: F(3, 48) = 0.928, p = 0.453 (Wilks’ Lambda).

In the **RDCP** group, the analysis showed no significant main effect of trial: F(3, 39) = 0.988, p = 0.434 (Wilks’ Lambda).

### Time spent on contact with the tunnels in the unchanged zone of the chamber

In the **ADD** group, the analysis showed a significant main effect of trial: F(3, 36) = 19.684, p < 0.001, Eta^2^ = 0.855 (Wilks’ Lambda). A post-hoc analysis showed a significant decrease in the time spent on contact with the tunnels in the unchanged zone in all test trials compared to the habituation phase (T1: p < 0.001, M_H_ = 80.74, SD_H_ = 16.47, M_T1_ = 46.62, SD_T1_ = 27.43; T2: p < 0.001, M_T2_ = 50.15, SD_T2_ = 18.20; T3: p = 0.001, M_T3_ = 47.08, SD_T3_ = 23.23).

In the **RMVL** group, the analysis showed no significant main effect of trial: F(3, 48) = 0.928, p = 0.453 (Wilks’ Lambda).

In the **RDCP** group, the analysis showed a significant main effect of trial: F(3, 39) = 8.263, p = 0.004, Eta^2^ = 0.693 (Wilks’ Lambda). A post-hoc analysis showed a significant increase in the time spent on contact with the tunnels in the unchanged zone in the third test trial (T3: p = 0.004, M_H_ = 75.93, SD_H_ = 26.57, M_T3_ = 113.43, SD_T3_ = 33.61).

### Frequency of contact with the tunnels in the unchanged zone of the chamber

In the **ADD** group, the analysis showed a significant main effect of trial: F(3, 36) = 7.312, p = 0.009, Eta^2^ = 0.709 (Wilks’ Lambda). A post-hoc analysis showed a significant decrease in the frequency of contact with the tunnels in the unchanged zone in all test trials compared to the habituation phase (T1: p = 0.002, M_H_ = 6.56, SD_H_ = 1.21, M_T1_ = 4.46, SD_T1_ = 1.85; T2: p = 0.039, M_T2_ = 5.17, SD_T2_ = 1.99; T3: p = 0.001, M_T3_ = 4.23, SD_T3_ = 1.24).

In the **RMVL** group, the analysis showed no significant main effect of trial: F(3, 48) = 1.467, p = 0.266 (Wilks’ Lambda).

In the **RDCP** group, the analysis showed no significant main effect of trial: F(3, 39) = 1.562, p = 0.254 (Wilks’ Lambda).

### Time spent on contact with the tunnels in the changed zone of the chamber

In the **ADD** group, the analysis showed a significant main effect of trial: F(3, 36) = 49.001, p < 0.001, Eta^2^ = 0.936 (Wilks’ Lambda). A post-hoc analysis showed a significant increase in the time spent on contact with the tunnels in the changed zone in all test trials compared to the habituation phase (T1: p < 0.001, M_H_ = 69.74, SD_H_ = 15.71, M_T1_ = 168.23, SD_T1_ = 36.63; T2: p < 0.001, M_T2_ = 161.15, SD_T2_ = 61.16; T3: p < 0.001, M_T3_ = 188.08, SD_T3_ = 43.85).

In the **RMVL** group, the analysis showed no significant main effect of trial: F(3, 48) = 0.701, p = 0.567 (Wilks’ Lambda).

In the **RDCP** group, the analysis showed a significant main effect of trial: F(3, 39) = 4.448, p = 0.028, Eta^2^ = 0.548 (Wilks’ Lambda). A post-hoc analysis showed a significant increase in the time spent on contact with the tunnels in the changed zone in the first test trial (T1: p = 0.025, M_H_ = 87.76, SD_H_ = 24.19, M_T1_ = 112.79, SD_T1_ = 31.77), and then a decrease in the third trial (T3: p = 0.030, M_T3_ = 69.86, SD_T3_ = 22.06).

### Frequency of contact with the tunnels in the changed zone of the chamber

In the **ADD** group, the analysis showed no significant main effect of trial: F(3, 36) = 2.542, p = 0.115 (Wilks’ Lambda).

In the **RMVL** group, the analysis showed no significant main effect of trial: F(3, 48) = 1.084, p = 0.388 (Wilks’ Lambda).

In the **RDCP** group, the analysis showed a significant main effect of trial: F(3, 39) = 7.673, p = 0.005, Eta^2^ = 0.677 (Wilks’ Lambda). A post-hoc analysis showed a significant increase in the time spent on contact with the tunnels in the changed zone in the first test trial (T1: p = 0.001, M_H_ = 6.10, SD_H_ = 2.28, M_T1_ = 8.36, SD_T1_ = 2.50). Table 1 shows descriptive statistical data on all the behavioural measurements taken in this study.

**Table 1:** Descriptive statistics of all behavioural measurements analysed in this study.

GroupsN=	ADD 13		RMVL 17		RDCP 14	
	Mean	Std Dev	Mean	Std Dev	Mean	Std Dev
**Latency to leave the transporter**						
Habituation trials	3.56	1.40	4.94	2,47	4.05	1.69
Trial T1	3.69	2.78	5.12	2.26	3.79	2.99
Trial T2	2.62	1.26	4.29	2.20	4.21	2.64
Trial T3	3.85	2.91	4.71	2.62	3.36	1.74
**Time spent in the transporter (excluding latency)**						
Habituation trials	63.62	19.93	54.63	14.84	51.10	15.06
Trial T1	36.62	20.54	60.65	18.68	37.00	20.04
Trial T2	41.31	26.18	49.65	24.83	58.79	37.31
Trial T3	34.54	21.93	83.59	52.93	44.64	23.56
**Time spent in the unchanged zone of the chamber**						
Habituation trials	131.54	15.83	118.00	32.97	122.38	39.99
Trial T1	68.00	20.78	141.18	63.74	113.79	48.6.0
Trial T2	99.85	42.38	140.00	62.97	118.29	33.82
Trial T3	82.23	37.19	123.88	54.85	163.29	49.29
**Time spent in the changed zone of the chamber**						
Habituation trials	117.62	18.31	136.12	43.34	135.40	34.93
Trial T1	220.62	38.85	124.29	34.19	158.07	30.94
Trial T2	210.85	72.11	123.00	63.95	130.29	38.50
Trial T3	231.85	48.37	117.12	49.80	113.21	27.89
**Frequency of moving between the zones (left/right/transporter) of the chamber**						
Habituation trials	14.18	2.15	14.59	2.06	15.10	4.60
Trial T1	12.62	2.75	13.82	2.77	15.71	4.55
Trial T2	11.77	1.96	13.94	2.86	16.36	3.82
Trial T3	12.38	2.43	13.41	4.15	14.64	4.16
**Time spent on contact with the tunnels in the unchanged zone of the chamber**						
Habituation trials	80.74	16.47	83.33	27.33	75.93	26.57
Trial T1	46.62	27.43	97.00	52.56	79.43	38.61
Trial T2	50.15	18.20	103.88	52.63	82.00	28.02
Trial T3	47.08	23.23	94.12	49.04	113.43	33.61
**Frequency of contact with the tunnels in the unchanged zone of the chamber**						
Habituation trials	6.56	1.21	5.57	1.70	6.40	2.95
Trial T1	4.46	1.85	6.24	1.68	5.71	2.67
Trial T2	5.17	1.99	5.94	1.82	5.50	2.24
Trial T3	4.23	1.24	4.94	2.61	6.86	2.48
**Time spent on contact with the tunnels in the changed zone of the chamber**						
Habituation trials	69.74	15.71	91.43	28.96	87.76	24.19
Trial T1	168.23	36.63	94.88	27.58	112.79	31.77
Trial T2	161.15	61.16	93.12	52.57	85.14	24.96
Trial T3	188.08	43.85	80.47	34.35	69.86	22.06
**Frequency of contact with the tunnels in the changed zone of the chamber**						
Habituation trials	5.49	0.85	5.80	1.35	6.10	2.28
Trial T1	7.08	1.80	5.24	1.48	8.36	2.50
Trial T2	6.31	2.78	5.76	1.99	6.79	2.04
Trial T3	6.85	2.30	6.12	1.41	5.29	1.49

#### Effect size analysis

Despite some limitations, partial eta squared (Eta^2^) is regarded as an effect size measure, which makes it possible to compare the results obtained in different subject groups or even studies^27^. Therefore, we decided to use this measure in order to obtain more detailed information about the effects of environmental change on rat behaviour in the experimental cage. Table 2 shows all the dependent variables collected in this study across the three experimental groups together with the Eta^2^ values.

**Table 2:** The ranking list of statistically significant effects based on the partial Eta^2^ values. The Eta^2^ values of statistically non-significant effects have been set to “0”.

Variable	Group	Eta^2^
Statistically significant effects		
Time spent on contact with tunnels in the changed zone of the chamber	ADD	0.936
Time spent in the changed zone of the chamber	ADD	0.889
Time spent on contact with the tunnels in the unchanged zone of the chamber	ADD	0.855
Frequency of contact with the tunnels in the unchanged zone of the chamber	ADD	0.709
Time spent on contact with the tunnels in the unchanged zone of the chamber	RDCP	0.693
Frequency of contact with the tunnels in the changed zone of the chamber	RDCP	0.677
Time spent in the transporter	ADD	0.666
Frequency of moving between the zones (left/right/transporter) of the chamber	ADD	0.633
Time spent in the unchanged zone of the chamber	RDCP	0.561
Time spent on contact with the tunnels in the changed zone of the chamber	RDCP	0.548
Time spent in the unchanged zone of the chamber	ADD	0.540
Time spent in the transporter	RMVL	0.219
**Statistically non-significant effects**		
Time spent in the transporter	RDCP	0
Time spent in the unchanged zone of the chamber	RMVL	0
Time spent in the changed zone of the chamber	RMVL	0
Time spent in the changed zone of the chamber	RDCP	0
Frequency of moving between the zones (left/right/transporter) of the chamber	RMVL	0
Frequency of moving between the zones (left/right/transporter) of the chamber	RDCP	0
Time spent on contact with the tunnels in the unchanged zone of the chamber	RMVL	0
Frequency of contact with the tunnels in the unchanged zone of the chamber	RMVL	0
Frequency of contact with the tunnels in the unchanged zone of the chamber	RDCP	0
Time spent on contact with the tunnels in the changed zone of the chamber	RMVL	0
Frequency of contact with the tunnels in the changed zone of the chamber	ADD	0
Frequency of contact with the tunnels in the changed zone of the chamber	RMVL	0

Kruskal-Wallis ANOVA (Eta^2^ value by group) clearly showed (H = 10.873, df = 2, p = 0.004) that the effects associated with the ADD group ranked significantly higher than the rest of the effects found in the GLM analysis.

#### Summary of the results

Group ADD: Rats from this group responded to the experimental manipulation (introduction of new tunnels) with a profound behavioural shift towards the newly installed tunnels. This effect was manifested by a significant increase in the time spent in the changed zone of the chamber, as well as the duration of contacts with the tunnels in the changed zone of the chamber. Consequently, for rats from this group, there was a decrease in the amount of time spent in the unchanged zone, in the duration of contacts with the tunnels in this zone and the length of stay in the transporter. Moreover, there was a decrease in the frequency of moving between the zones and in the frequency of contacting the tunnels in the unchanged zone of the chamber.

Group RMVL: Rats from this group barely responded to experimental manipulation (removal of tunnels). The only response observed was their longer stay in the transporter.

Group RDCP: Rats from this group responded to the experimental manipulation (reduction of environmental complexity) in a complex way. In the first test trial, there was an increase in the duration and frequency of contacts with the tunnels in the changed zone. However, in the third test trial, the rats spent more time in the unchanged zone, as well as on contact with the tunnels in that zone.

## Discussion

The purpose of this study was to explore the responses to novelty which are triggered by changes in the stimulus field as a result of increased and decreased complexity. The environmental changes involved introducing novel objects (tunnels), removing objects or changing the properties of the existing objects in a way that decreased environmental complexity. The discussion of the results will be based on the following analytical elements: the environmental change (experimental manipulation), direction of the behavioural response and the effect size analysis.

The experimental manipulations showed differential effects on the rats’ behaviour in the test box. Adding new tunnels triggered a very profound change in behaviour, which was demonstrated by the animals’ prolonged stay in the proximity of the novel objects, sniffing, touching, and climbing on top of the tunnels. This was reflected in the following measurement results: the amount of time spent on contact with the tunnels in the right zone of the chamber, the frequency of contact with the tunnels in the right zone of the chamber and the amount of time spent in the right (changed) zone of the chamber. The ADD rats reduced their activity in the left zone of the chamber and in the transporter. The analysis of the effect size also showed that Eta^2^ estimations were the highest in this group. This activity shift towards the newly introduced objects in familiarized surroundings replicates and confirms the results of previous studies^16–20,29,30^, in which this manipulation technique (addition of new tunnels) had also been utilized. This effect is in line with the more general observations on the reinforcing value of novel stimulation, as proposed by Pisula^27^ (p.101).

The next manipulation ‒ the removal of the tunnels from the test arena ‒ turned out to have the least influence on behaviour compared to the other manipulations used in this study. This is somewhat incompatible with the results of an earlier study by Pisula and Siegel^17^, where both the addition and the removal of the tunnels had had comparable effects on the rats’ behaviour. However, some differences in the measurement technique, such as the software used in this study and the focus on the animal-environment interaction (and not on the precise form of behaviour) may be considered as causal factors in the observed differences in findings. The results of this study suggest that the removal of familiarized objects has much less influence on the behavioural response than the introduction of novel objects. The only effect of the RMVL procedure was the increase in the time spent in the transporter in the last test trial. This may reflect a gradual decrease in the attractiveness of the changed zone of the chamber, which lead to allocating more time to the comfortable and safe space provided by the transporter.

The last (but no less significant) manipulation involved a procedure that combined keeping the overall arrangement of the test area unchanged, while decreasing the internal complexity of the tunnel in the right zone of the experimental box. This was achieved by building a substitute for the four-tunnel construction (which had previously been used in the manipulations) that consisted only of the external surface elements and which was hollow inside. This environmental change turned out to be of intermediate strength compared to the most powerful ADD manipulation, and the least influential RMVL procedure. The modification of the test area may be described as a reduction in the complexity of the object that continued to be present in the chamber. This might be regarded as a more subtle modification than the addition or removal of entire objects. This change, albeit less evident than the removal or addition of entire objects, still produces a decrease in environmental complexity in terms of affordances^1,23^. If the perceptual system is predisposed to associate the perceived objects with specific motor actions, then the rats may be driven to adapt to a new number of possible actions within the test area. Since that change in the properties is less obvious, as compared, for instance, with the situation where the object is removed, this adaptation requires more physical and cognitive effort on the part of the individual. This phenomenon may be directly linked to the properties of the objects which may be affordant to a larger or lesser extent^31^. As argued by Heyser and Chemero^31^, animal responses to novel objects could ‒ and should ‒ be described in terms of the functional (affordant) properties of the various objects. The authors of this paper fully agree with that claim.

The results of this study clearly show that the properties of the novel stimulation are of crucial importance for the rats’ behavioural regulation. In itself, novelty only accounts for a fraction of the regulating mechanisms. The combination of novelty and change in the level of available affordances results in the final level of behavioural effort that an animal needs to adapt to those environmental changes. Changes in the environment that open new possibilities (affordances), both due to the addition of novel objects and because of an increase in environmental complexity, require the highest level of behavioural and cognitive effort necessary to incorporate these changes into the cognitive system. In this study, changes of this kind were introduced in the ADD procedure; they may be described as the most ‘affordance-inviting’, to use the term coined by Withagen, de Poel, Araújo and Pepping^32^.

On the other hand, the RMVL procedure created an environmental change that did not demand any exploratory effort. Therefore, the animals in this group made the least effort when adapting to this situation. It seems that the preliminary exploration of the modified part of the test arena allowed the animals to incorporate the changes into their perception of the surroundings. The drop in the available affordances or environmental complexity (reflected in the number of available objects), may be described as an ‘affordance-disinviting’ situation.

It seems interesting to consider the possible consequences that this type of reaction to a specific change occurring in the environment may have for the animals’ development. It may be hypothesised, based on time-energy budget analyses^33^, that investing time and energy in the exploration of novel objects is much more effective, than devoting efforts in investigating space from which the object disappeared. Therefore, it is possible, that the evolution of the cognitive system has led to the development of higher cognitive sensitivity to the emergence of novel objects (potentially hazardous or beneficial) rather than to the loss of some environmental features [cf.^34^]. Some other concurrent explanations should also be considered. Comparative studies involving ecological factors are necessary to shed further light on this topic.

The RDCP procedure combined keeping the overall arrangement of the test area unchanged, while decreasing the internal complexity of the tunnels in the right chamber zone. In terms of environmental properties, this manipulation was similar to some extent to the RMVL procedure. However, this procedure turned out to be more demanding for the animals than the RMVL procedure in terms of the amount of effort needed for adaptation. It seems that modifications which are not evident (not recognizable during the initial investigation) produce a behavioural change that is directed towards the source of that modification. This was seen in the first test trial, during which the animals were able to recognize the modification that had been done to their environment. Consequently, as they had recognized the drop in affordances, the rats did not spend much time and did not make much exploratory effort in this zone in the second and third test trials. The change in the rats’ activity from the changed zone to the unchanged zone, which was observed in the course of the three test trials, may be regarded as a change in the dominant mechanisms of behaviour regulation. First, the novelty resulting from environmental change triggers exploratory activity. This was manifested in the proximity of the source of novelty, i.e. the changed tunnels. However, after having processed the sensory input, the rats switch to behaviour regulation mechanisms based on the cross-situational stable preferences, e.g. preference for complexity, and moved towards the more complex zone of the test arena. The transition process in itself and the mechanisms underlying it are an interesting subject for future comparative analyses.

Tunnels are important elements in the rats’ environment, since they provide various possibilities for hiding, resting or moving inside the tunnel. They may be treated as a good example of affordances in rat-environment interactions. The results of this study may therefore provide a basis for constructing a modified theory of animal curiosity which could incorporate the concept of ecological psychology. Since we are open to the possibility of parallel and/or convergent evolution of cognitive adaptations in various animal taxons^35^, it would be worthwhile to exploit this phenomenon from the perspective of comparative psychology and conduct a study of various species, including humans.

Reaction to changes in environmental complexity, including reactions to new objects introduced to familiar environments, has been studied in many fields of psychology. Our study may provide new data concerning the interpretation of the results e.g. of the Novel Object Recognition (NOR), e.g.^36^, which is commonly used for measuring memory impairments occurring in various neuropsychiatric disorders. The introduction of objects of varying complexity and the removal of objects from the experimental arena is likely to trigger different behavioural reactions in the test animals. In addition, the impact of novelty and environmental complexity on the nervous system, and, in consequence, on behaviour, is particularly significant in the context of environmental enrichment (e.g^11,37^). Our study shows that when manipulating the type of enrichment, special attention must be paid not only to the changes introduced, but also to the level of complexity of those changes. A change involving environmental impoverishment should not be regarded as environmental enrichment. The differential response to novelty produced by the emergence of new objects/qualities, as opposed to novelty resulting from their removal, might be also interesting from the point of view of evolutionary and clinical human psychology. The impact of this effect, however, has to be confirmed by further studies.
